# OS‐01 Peptide Topical Formulation Improves Skin Barrier Function and Reduces Systemic Inflammation Markers: A Pilot 12‐Week Clinical Trial

**DOI:** 10.1111/jocd.70169

**Published:** 2025-04-07

**Authors:** Alessandra Zonari, Lear E. Brace, Luiza Brunelli Buhrer, Nathaniel H. O. Harder, Claire Harker, Adam B. Aronson, Caitlyn N. Tse, Carolina R. Oliveira, Mariana Boroni, Juliana L. Carvalho

**Affiliations:** ^1^ OneSkin, Inc. San Francisco California USA; ^2^ Skin Cancer and Reconstructive Surgery Center Newport Beach California USA; ^3^ Bioinformatics and Computational Biology Lab Brazilian National Cancer Institute (INCA) Rio de Janeiro RJ Brazil; ^4^ Genomic Sciences and Biotechnology Program Catholic University of Brasilia Brasília DF Brazil; ^5^ Laboratory of Interdisciplinary Biosciences, Faculty of Medicine University of Brasília Brasília DF Brazil

**Keywords:** clinical trial, inflammation, senescence, skin aging, skin barrier function

## Abstract

**Objective:**

As the body's largest organ, the skin plays a crucial role in defending against external stressors. Skin characteristics change with age, decreasing skin barrier integrity and compromising skin and body health. This study aimed to investigate the potential of a topical formulation containing OS‐01 (a.k.a. Peptide 14), a senotherapeutic peptide, to counteract age‐related skin changes and their systemic consequences.

**Methods:**

A randomized, double‐blinded clinical trial involving 60 female volunteers aged 60–90 was conducted over 12 weeks. Participants received either an OS‐01 topical formulation or a commercially available moisturizer control formulation. Skin parameters, subjective perceptions, and circulating cytokine levels were assessed. Skin instrumental analysis included transepidermal water loss (TEWL), skin hydration, and pH measurements.

**Results:**

Participants treated with the OS‐01 topical formulation displayed significantly improved skin barrier function and hydration compared to the control group. Participant perceptions aligned with objective findings: after 12 weeks, 70% of participants in the OS‐01 group noticed an improvement in general skin appearance versus 42% for the control group. The systemic levels of proinflammatory cytokines tended to normalize, with a significant decrease in IL‐8 in the blood analysis of participants from the OS‐01 group. On the other hand, the control group demonstrated an increase in a few circulating cytokines, particularly TNF‐ɑ and IFN‐γ. Moreover, GlycanAge analysis measuring participants' biological age suggested the slowing of systemic aging in the group treated with the OS‐01 topical formulation.

**Conclusion:**

The study suggests that the OS‐01 formulation can impact skin health by improving the skin barrier function, potentially influencing systemic inflammation and biological age. In conclusion, the study supports that targeting skin health may contribute to better longevity outcomes, underscoring the skin's pivotal role in systemic aging and supporting an integrated approach to health management.

## 1 Introduction

The skin is the body's largest organ, and it plays a critical role in its defense against external factors, including chemical, radiological, and biological stressors. However, research into the role of skin aging is often correlated strictly with skin appearance without considering changes in the pathobiological basis and consequences of functional decline. Recent studies show that aged skin is a product of intrinsic and extrinsic factors that act synergistically to impose genetic, epigenetic, proteomic, metabolic, and microbiomic changes that lead to the functional decline of the tissue [1].

As the human body ages, complex changes in the skin's microenvironment weaken its immune defenses and overall functionality, increasing its vulnerability to damage and disease [2, 3]. Notably, while aging is associated with a loss of Langerhans cells in the skin, the prevalence of mast cells and T cells escalates, especially in sun‐exposed regions [1]. Macrophage function also becomes compromised and increasingly proinflammatory [4]. Immunosenescence results from the accumulation of senescent cells and impedes an effective response to infections and cancer [5]. Skin barrier function and maintenance also decline, fueled by exhausted stem cell pools [6], resulting in increased skin fragility and longer healing time [7].

The aged and atrophic skin barrier, combined with dysfunctional immune and microbiome components, is reflected in increased senescent cell populations and the corresponding senescence‐associated secretory phenotype (SASP) secretion components, notably a proinflammatory state [8]. Clinically, increased rates of skin inflammatory disorders and opportunistic infections are observed in the elderly [3, 9].

Recent evidence suggests that the accumulation of cellular senescence in the skin may drive further consequences of systemic aging, such as chronic inflammation [10, 11, 12] and immunosenescence [13], both of which have been shown to accelerate biological aging [14]. Those events cause and/or aggravate chronic diseases, such as cancer [15], cardiovascular diseases [16], autoimmune, and metabolic disorders [17]. It also increases the risk of mortality [18]. As the largest organ in our body, it does not come as a surprise that its dysfunction affects other bodily systems.

Currently, one preclinical study [19] and one pilot clinical study [20] have demonstrated that the age‐related deterioration of skin function constitutes an actionable target to decelerate systemic inflammation. OS‐01 (a.k.a Peptide 14) is a senotherapeutic peptide that is proven to be safe [21], to strengthen the skin barrier effectively, and to reduce cellular senescence and skin's biological age in human skin models [22]. Additionally, a double‐blind, vehicle‐controlled clinical study confirmed that the topical application of the OS‐01 peptide to the face can improve skin barrier function by reducing transepidermal water loss measurement [23]. In this study, we conducted a randomized, double‐blind clinical trial on aged volunteers to test the effectiveness of a topical formulation containing the OS‐01 peptide compared to a control emollient formula previously shown to support skin barrier function and to reduce the plasmatic levels of inflammatory molecules [20]. We evaluated the skin quality, skin barrier function, circulating inflammatory profile, and biological age before and after 12 weeks of treatment.

## 2 Methods

### 2.1 Clinical Study Design

The study is a randomized, double‐blinded, 12‐week, prospective study that evaluates the effect of two topical treatments on skin parameters and circulating cytokine levels of older volunteers. Before initiation, the study was approved by the Veritas Institutional Review Board (2022‐3038‐10 910‐5). The study was carried out in accordance with the principles set forth by the Declaration of Helsinki. Informed consent was obtained from all participants.

Sixty female volunteers over the age of 60 were recruited according to specific inclusion and exclusion criteria (Table S1), and divided into two experimental groups. Following informed consent, their skin was assessed by instrumental analysis to determine baseline levels of skin barrier function, hydration, and pH level, and a blood sample was drawn to measure circulating cytokine levels. After this stage, the participants were randomly assigned to one of two groups: group A (*n* = 30, final *n* = 25) received unidentified bottles containing a control formulation (Atopalm MLE Lotion, Neopharm, South Korea). This formula has been previously shown to strengthen the epithelial barrier and reduce inflammatory mediators in the plasma of aged volunteers [20]. Group B (*n* = 30, final *n* = 27) received unidentified bottles containing OS‐01 peptide topical formulation (OS‐01 BODY, OneSkin, USA). The list of ingredients for each formulation is shown in Table S2. Participants' demographics are shown in Table 1. Five participants from group A dropped out of the study due to inconsistent application, extended vacation, lack of follow‐up, and stress unrelated to the study. For group B, the three dropouts were due to moderate adverse skin reactions, including persistent rash/pimples and dry patches, possibly attributed to the product or to the provided cleanser.

**TABLE 1:** Subject demographics that concluded the study.

Group	No. of participants	Age range	Average age	Gender
Control formulation	25	61–85	72.43 ± 6.84	Female
OS‐01 Formulation	27	62–82	69.96 ± 5.48	Female

**FIGURE 1:**
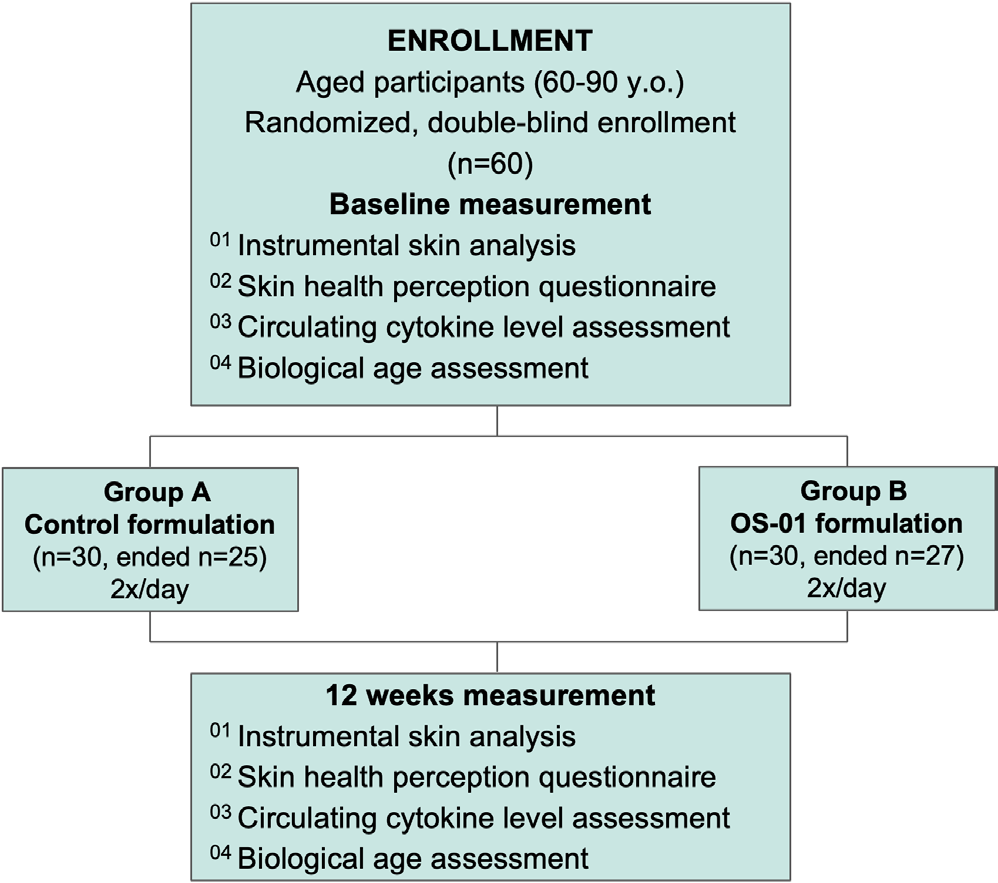
Flowchart illustrating the subject enrollment and treatment.

All volunteers were instructed to apply the creams to the body twice daily for 12 weeks. Additionally, to minimize variations from differing skincare routines, a support product consisting of a body cleanser (Cetaphil Ultra Gentle Body Wash, Galderma Laboratories, Germany) was supplied, and it was common between groups A and B. Participant group randomization was executed in a way to maintain participants and researchers blinded to the experimental group. Treatment adherence was verified by monthly online monitoring. Questionnaires were utilized to assess the volunteers' perception of their skin at baseline, 4 weeks, and 12 weeks. After 12 weeks of use, participants returned, and instrumental measurements and blood collection were repeated. A flowchart is presented in Figure 1, illustrating the experimental groups and the executed analysis.

### 2.2 Skin Instrumental Analysis

Participants from groups A (control) and B (OS‐01) had their skin analyzed using three different sensors, in order to quantify different parameters related to skin health. All measurements were obtained from both the right and left arms, and results were averaged.

#### 2.2.1 Skin Barrier Function Assessment

Transepidermal Water Loss (TEWL) was determined using a Tewameter TM 300 MDD (Acaderm). Changes in TEWL provide a measure of skin barrier function, as well as a disruption or compromised integrity of such a barrier. The lower the TEWL values, the higher the skin barrier function.

#### 2.2.2 Skin Hydration

Quantified using a Corneometer CM 825 (Acaderm). Changes in skin capacitance reflect alterations in skin hydration levels. The higher the corneometer values, the better the skin's hydration levels.

#### 2.2.3 Skin pH

Assessed using a Skin pH‐Meter PH 905 (Acaderm). An optimal range in skin pH, between 4.7 and 5.8, is associated with balanced skin function and microbiome.

### 2.3 Subjective Questionnaires

Subjective questionnaires were used to gauge the subjects' perception of their skin and the effect of the applied products. Subjects assessed noticeable changes related to aging and skin quality using five‐point scales (1 being the least noticeable effects and 5 being the most noticeable effects). The percentage of subjects who noticed improvement was determined based on answers above 3.

### 2.4 Blood Collection and Measurement of Plasma Cytokines, GlycanAge, and OS‐01 Peptide Levels

At the beginning and conclusion of the 12‐week study period, participants from both groups (Control and OS‐01) underwent blood sampling. For this, two 3 mL blood samples were collected from each participant. Blood was drawn into EDTA tubes, which prevent clotting and preserve the blood constituents. The samples were then immediately centrifuged to separate plasma, which was subsequently frozen at −80°C.

#### 2.4.1 Cytokine Measurement

The plasma samples were analyzed for a comprehensive cytokine profile. This analysis was conducted by Eve Technologies Corporation (Canada) using a high‐sensitivity multiplex assay (Array HDF15). This assay allowed for the simultaneous quantification of various cytokines, including GM‐CSF, IFNγ, IL‐1β, IL‐1RA, IL‐2, IL‐4, IL‐5, IL‐6, IL‐8, IL‐10, IL‐12(p40), IL‐12(p70), IL‐13, MCP‐1, and TNFα. These cytokines were selected due to their relevance in signaling pathways associated with inflammation, immune response, and cellular senescence. The data are presented as mean ± SEM for each cytokine at baseline and after 12 weeks. However, for cytokines GM‐CSF, IL‐2, IL‐4, IL‐12(p70), and IL‐13, the majority of the samples were out of range; hence, no data were presented.

#### 2.4.2 GlycanAge Analysis

Alongside cytokine analysis, plasma samples were sent to GlycanAge Ltd. (Croatia) for biological age determination. GlycanAge assesses biological aging by analyzing glycan structures on immunoglobulin G (IgG). Glycans attached to IgG change with age and can reflect the state of the immune system and inflammation. The GlycanAge test involves analysis of these glycan structures, providing an index that correlates with an individual's biological age [24]. This index was determined for each participant, both at baseline and after the 12‐week treatment period.

#### 2.4.3 OS‐01 Peptide Assessment by Mass Spectrometry

Mass spectrometry was used to detect the presence of the OS‐01 peptide in the plasma of participants in Group B. An aliquot of 20 μL of human plasma was extracted with 100 μL of methanol: acetonitrile (5:95, v:v) containing the internal standard (verapamil). The mixture was shaken for 15 min and subsequently centrifuged at 3220 *g* for 15 min. An aliquot of 70 μL of the supernatant was mixed with 70 μL of water with 0.1% formic acid for injection to the LC–MS. Calibration standards and quality control samples were prepared by spiking the test compound into blank human plasma and then processed with the unknown samples in the same batch. An ExionLC AD interfaced to a Qtrap 6500+ system from Sciex (Redwood City, CA) was used for bioanalysis. The samples were injected onto an Aeris Widepore C4 column (2.1 × 100 mm, 5 μm) from Phenomenex (Terrance, CA) and separated by gradient elution using water with 0.1% formic acid (A) and acetonitrile with 0.1% formic acid (B) as mobile phases. The gradient program started at 3% B, held for 0.2 min, ramped to 85% B at 1.2 min, remained at 85% until 1.8 min, returned to 3% B at 1.85 min, and stopped at 2.3 min at a flow rate of 0.7 mL/min. The mass spectrometer was operated in positive electrospray ionization under multiple reaction monitoring (MRM) mode for the detection of the analyte, peptide 14 (395.3‐to‐129.0 *m*/*z*), and the internal standard, verapamil (455.3‐to‐165 *m*/*z*). The calibration curve fitted by linear regression with a weighting factor of 1/X2 was used to quantify the analyte concentrations in the matrix using Analyst software 1.7.0 from Sciex.

### 2.5 Statistical Analysis

Data from instrumental analysis are expressed as mean ± SD. The remaining data are expressed as the mean ± SEM. Data were analyzed using GraphPad Prism 9 software for statistical analyses. A paired *t*‐test was used to determine the differences in each instrument analysis, cytokine level, and GlycanAge between baseline and 12‐week treatment. An unpaired t‐test with Welch's correction was used to assess the significance of differences between the control and OS‐01 groups for the delta age analysis. Significant data were considered when *p* < 0.05.

## 3 Results

### 3.1 Skin Instrumental Analysis

Instrumental analysis was performed after 12 weeks on the forearm and upper arm as these are commonly exposed and visible areas of the body. Participants in both OS‐01 and control groups exhibited significant enhancement in skin barrier function, as quantified by transepidermal water loss (TEWL) using a tewameter. Participants from the OS‐01 group demonstrated a significant improvement in TEWL of 41.49% on the forearm (*p* = 0.001) and 34.73% on the upper arm (*p* = 0.027). In comparison, the participants in the control group exhibited a 40.00% significant improvement only on the forearm (*p* < 0.001) but not significant on the upper arm, with a 24.24% improvement (*p* = 0.090) (Table 2).

**TABLE 2:** Instrument analysis.

Area of treatment	Instrument	Time point	Control formulation					OS‐01 formulation					*p* value Ctrl vs. OS
			*n*	Mean ± SD	Mean percent improvement from BL mean	Percent of subjects showing improvement from BL	*p* value 12 weeks vs. BL	*n*	Mean ± SD	Mean percent improvement from BL mean	Percent of subjects showing improvement from BL	*p* value 12 weeks vs. *BL*	
Forearm	Tewameter	Baseline	25	5.68 ± 2.83				27	6.07 ± 4.23				
		12 weeks	25	3.32 ± 1.16	**40.00%**	**76.0%**	**< 0.001**	27	3.55 ± 1.81	**41.49%**	**77.8%**	**0.001**	0.924
	Corneometer	Baseline	25	27.73 ± 14.17				27	23.92 ± 13.29				
		12 weeks	25	32.15 ± 11.94	**15.32%**	**68.0%**	**0.048**	27	33.09 ± 11.59	**38.34%**	**81.5%**	**< 0.001**	0.075
	pH Meter	Baseline	25	5.85 ± 0.72				27	5.62 ± 0.53				
		12 weeks	25	5.57 ± 0.56	4.64%	72.0%	0.079	27	5.67 ± 0.93	NI	66.7%	0.8562	0.211
Upper Arm	Tewameter	Baseline	25	4.45 ± 2.52				27	5.87 ± 5.76				
		12 weeks	25	3.37 ± 1.43	24.24%	52.0%	0.090	27	3.83 ± 2.58	**34.73%**	**55.6%**	**0.027**	0.613
	Corneometer	Baseline	25	31.98 ± 10.33				27	25.39 ± 9.48				
		12 weeks	25	34.27 ± 10.82	7.16%	56.0%	0.363	27	35.14 ± 14.20	**38.39%**	**74.1%**	**0.002**	**0.022**
	pH Meter	Baseline	25	5.88 ± 0.58				27	5.87 ± 1.18				
		12 weeks	25	5.61 ± 0.51	**4.55%**	**72.0%**	**0.035**	27	5.72 ± 0.85	2.49%	70.4%	0.557	0.693

*Note:* The bold values are the significant values in which *p* > 0.05.

Corneometer assessment revealed substantial increases in skin hydration levels for the OS‐01 group, with a 38.34% improvement observed on the forearm (*p* < 0.001) and a 38.39% improvement on the upper arm (*p* = 0.002), as detailed in Table 2. Once again, a significant improvement (of 15.32%) in the control group was only observed on the forearm (*p* = 0.048) but not in the upper arm (7.16%, *p* = 0.363). Direct comparisons of the hydration improvement between participants in the control and OS‐01 groups reveal a significant enhancement in the hydration of the upper arm (*p* = 0.022) and close to significance in the forearm (*p* = 0.075) on the subjects using the OS‐01 formulation.

At baseline, all participants' skin pH was within the normal range, between 4.7 and 5.8. A significant reduction was only observed in the upper arms of the participants from the control group, changing from pH = 5.88 ± 0.58 to 5.61 ± 0.51 (*p* = 0.035). Although no significant change was observed in the OS‐01 group, the skin pH was kept within the normal range after 12 weeks of treatment (Table 2).

### 3.2 Participant Perception

**FIGURE 2:**
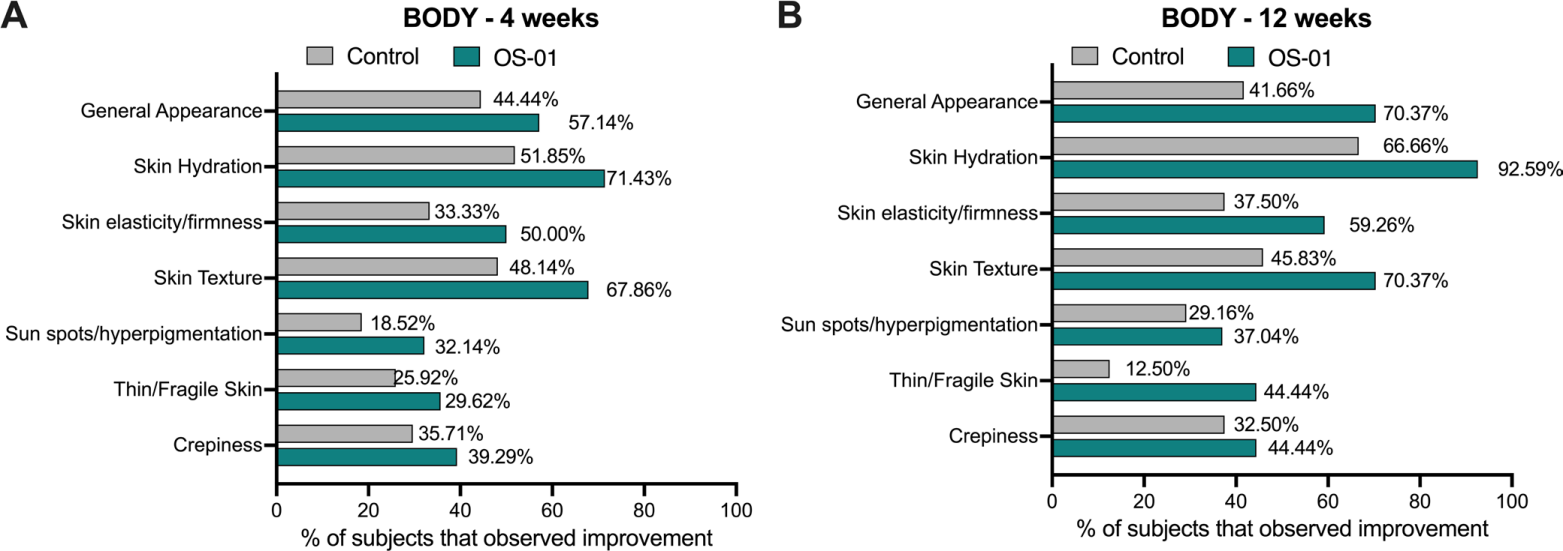
Subject questionnaire results for 4 (A) and 12 (B) weeks.

A self‐perception questionnaire was conducted to complement the objective findings from instrument analysis. Data were collected at baseline, week 4, and week 12. Data are represented as the percentage of subjects that notice improvement from the baseline perception. Participants from both groups reported noticeable improvement in various aspects of their skin. A higher percentage of subjects reported skin improvements in the OS‐01 group. Significant improvements in skin hydration, texture, elasticity/firmness, thin/fragile, and general appearance were observed, corroborating with the instrumental analysis (Figure 2). After 4 weeks, 71.43% of the OS‐01 group participants reported improvement in skin hydration, which increased to 92.59% of the participants after 12 weeks. These are superior numbers compared to the control group, which had 51.85% and 66.66% of the participants reporting positive effects after 4 and 12 weeks, respectively.

### 3.3 Plasma Cytokine Levels

**FIGURE 3:**
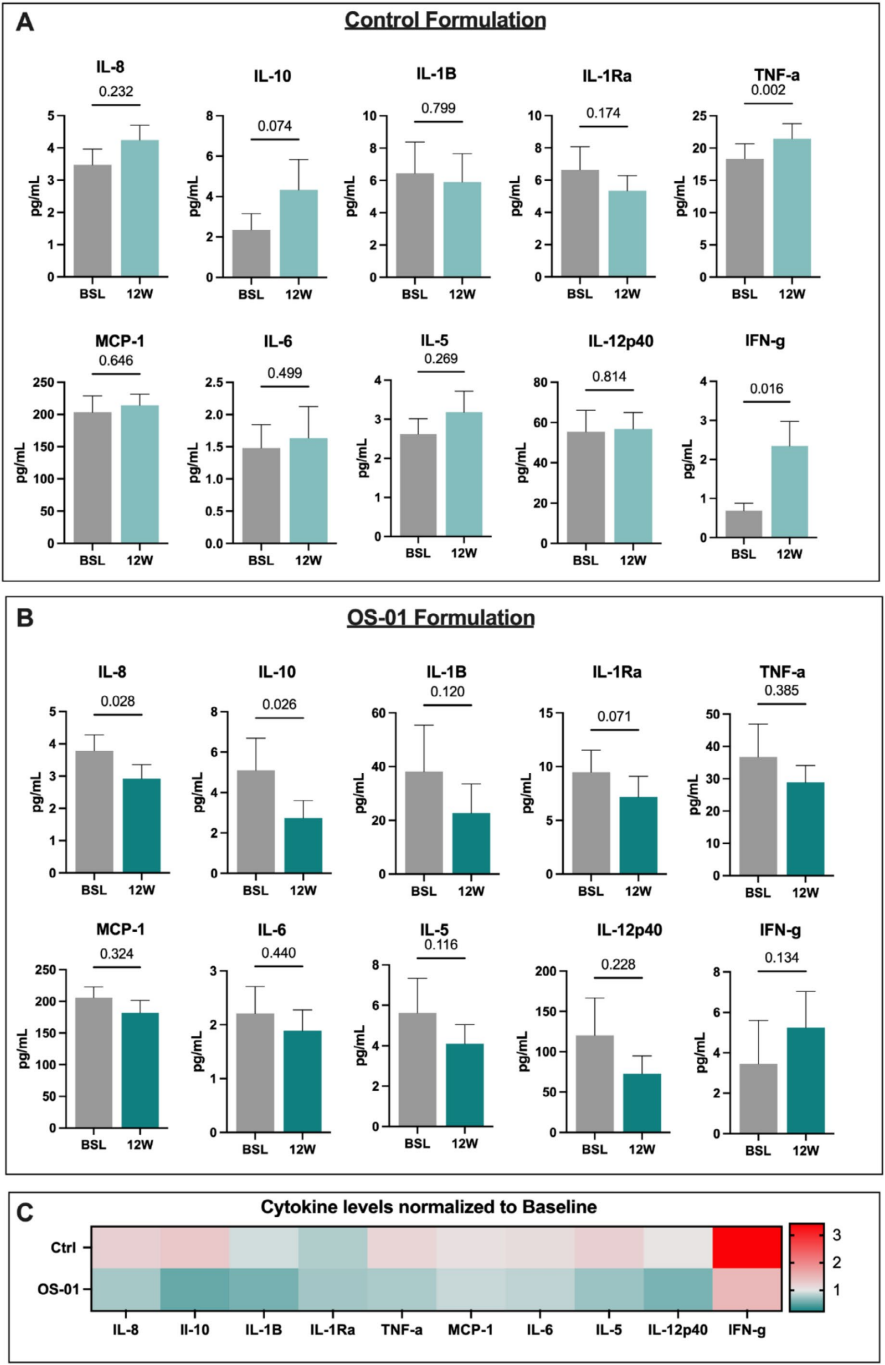
Circulating Levels of Inflammatory Cytokines at Baseline and 12 weeks after treatment. (A) control treatment and (B) OS‐01 treatment. BSL = baseline, 12 W = 12 weeks. Data are shown as mean ± SEM and were analyzed using paired *t*‐test. *p* values are indicated above each graph, and statistical significance was considered when *p* < 0.05. (C) Heatmap with the overall profile of circulating cytokines normalized to baseline.

Systemic cytokine levels were evaluated at baseline and after 12 weeks under the hypothesis that improvement in the skin barrier function could reduce systemic inflammation. Subjects using the OS‐01 formulation display decreased overall levels of inflammatory responsive biomarkers. In contrast, cytokine levels of participants treated with the control formula failed to decline and tended to be slightly elevated after 12 weeks, though only significantly increased for TNFα (*p* = 0.002) and IFN‐ɣ (*p* = 0.016) (Figure 3A). There is a global trend in participants of the OS‐01 group towards lower concentrations in all cytokines, except for IFN‐ɣ, which is increased but not significantly. Participants in the OS‐01 group showed significant decreases in IL‐8 (*p* = 0.028) and IL‐10 (*p* = 0.026) (Figure 3B). When visualized as a heatmap, the decline in circulating cytokine levels observed in the OS‐01 group (Figure 3C) implies that enhancing epidermal function has the potential to restore systemic cytokine levels to levels surpassing the baseline.

Mass spectrometry analysis of plasma from participants in the OS‐01 group showed no detection of the OS‐01 peptide at baseline or after treatment, indicating that the topical formula had only local effects on improving the skin barrier and promoting skin hydration (Table S3).

### 3.4 Assessment of Plasma IgG Glycan Degradation

**FIGURE 4:**
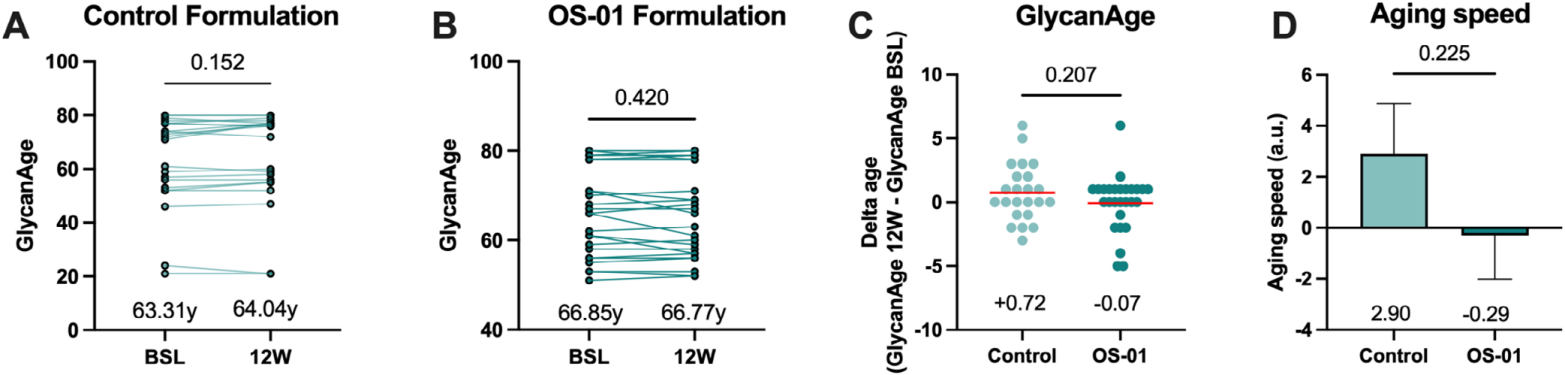
Determination of the Biological Age measured by changes in the IgG glycan. GlycanAge at baseline and after 12‐week treatment on participants treated with control (A) and OS‐01 formulation (B). *p* value was determined by paired *t*‐test. (C) Delta age measurement determined by the GlycanAge at 12 weeks minus GlycanAge at baseline. The red line represents the mean average. *p* value was determined by unpaired *t*‐test with Welch's correction. (D) Aging speed from control and OS‐01 groups, calculated by the change in age per individual divided by 0.25. Data are presented as mean ± SEM, and *p* value was determined using Unpaired *t*‐test with Welch's correction.

Immunoglobulin G (IgG) glycan changes have been associated with aging, as well as a host of pathologies linked to chronic inflammation. While the mechanisms and effects of these differential glycosylation patterns have yet to be fully elucidated, changes in IgG glycosylation can point to the relative age of an individual [24]. The plasma of the participants, collected at baseline and after 12 weeks, was analyzed for IgG glycan composition, and the biological age was determined (GlycanAge Ltd., Croatia). GlycanAge assessment revealed stabilization of aging in the OS‐01 group over the course of the study. In contrast, there was a trend towards increased aging in the control group (Figure 4A,B). Given a 12‐week time from baseline to study completion, subjects are expected to age by +0.25 years. However, the control appears to increase the age by an average of +0.72 years, while the participants treated with the OS‐01 formula present an average −0.07 year change (Figure 4C). This difference is also reflected in the aging speed, calculated by the change in the GlycanAge per individual divided by 0.25, as a measure of how quickly a participant would biologically age in a year with each treatment (Figure 4D). Compared to the control treatments, subjects in the OS‐01 group demonstrate a trend of slower systemic aging.

## 4 Discussion

To date, skin aging has been intricately linked to cellular senescence accumulation, chronic inflammation, and dysbiosis, three of the archetypal twelve Hallmarks of Aging [25, 26, 27]. Aged skin typically exhibits functional abnormalities, including compromised permeability barrier homeostasis, elevated stratum corneum pH, and reduced hydration, all factors that can contribute to proinflammatory cytokine secretion [28].

Previous studies using in vitro skin models have shown that the OS‐01 peptide supports skin health by mitigating cellular senescence, reducing DNA methylation age, enhancing DNA repair mechanisms, increasing epidermal thickness, and suppressing SASP‐associated gene expression [22]. Furthermore, clinical results from a double‐blind, split‐face, vehicle‐controlled study confirmed that the OS‐01 peptide, when applied topically to the face, helps improve skin barrier integrity by decreasing TEWL [23]. In this study, we hypothesized whether a body‐wide application of a topical formulation containing the OS‐01 peptide could improve skin barrier function, impacting systemic levels of inflammation. This topical formulation consists of an emulsion composed of different oils and, besides the OS‐01 peptide, other actives known to have anti‐inflammatory and antioxidant properties, including Sulforaphane (found in the *Lepidium Sativum* Sprout Extract), Genistein, and *Tremella Fuciformis* Sporocarp Mushroom Extract. The control group used a physiologic lipid‐containing emollient formula previously described to improve the skin barrier [20, 29].

Our investigation into the topical application of the OS‐01 peptide provides insights into the potential of targeting senescent cells and enhancing the skin's barrier to modulate systemic inflammatory responses. The tendency of declining cytokine levels within the OS‐01 cohort supports the hypothesis that cutaneous health mirrors and can influence systemic inflammation, which aligns with previous findings [19].

As expected, based on previous studies with the control formula and preliminary and mechanistic studies with OS‐01 treatments, there is an observed improvement in skin barrier function in both groups measured in the forearm. However, the OS‐01 formulation improves the skin barrier function more significantly than the control treatment. Significant improvement was observed in two different body areas (forearm and upper arm), while the control formulation effectively improved the skin of the forearm but not the upper arm. The hydration improvement was also superior in the OS‐01 group compared to the control group, most noticeably on the upper arm, where a significant improvement was observed in the OS‐01 group compared to the control. Our data corroborate previous findings in which this control formulation demonstrated improvement of the skin barrier and hydration on the forearm [20].

Subjective enhancements in skin quality reported by study participants complement the objective improvements noted through instrumental analysis. After 12 weeks, a greater percentage of participants in the OS‐01 group noticed an improvement in skin quality, including overall appearance, skin hydration, elasticity, texture, hyperpigmentation, thinning, and crepiness. Previous in vitro studies have shown the effects of the OS‐01 peptide on skin aging, including reduction in SASP, improvement in the epidermal thickness, and decrease of the skin's biological age [30]. We posit that these mechanisms may be the cause of the improved skin barrier function and hydration on the skin, which led to a better perception of the skin quality by the participants in the OS‐01 group.

Systemic benefits from the improvement of the skin barrier in the OS‐01 group were evident in circulating cytokine levels. Previous studies have correlated disrupted epidermal barrier function with increased serum inflammatory cytokines, which can contribute to a proinflammatory state thought to play a role in the pathogenesis of chronic systemic diseases [19]. These markers include IL‐1, IL‐1 receptor antagonist protein (IL‐1RA), IL‐6, IL‐8, IL‐13, IL‐18, C‐reactive protein (CRP), IFNα and IFNβ, transforming growth factor‐β (TGFβ), tumor necrosis factor (TNF), and its soluble receptors (TNF receptor superfamily members 1A and 1B), as well as serum amyloid A [31].

The participants in the OS‐01 group demonstrated an overall reduction in proinflammatory cytokines, with the mean average of the cytokines being reduced after 12 weeks compared to baseline, and with a statistically significant reduction of IL‐8 (*p* = 0.028) and trends towards reduction of IL‐1RA (*p* = 0.071). IL‐8 is a proinflammatory chemokine that activates inflammatory cells by the migration of neutrophils, mononuclear phagocytes, and mast cells and plays a major role in acquired immune responses. Elevated IL‐8 levels have been implicated in the pathogenesis of various age‐related diseases, including arthritis, Alzheimer's disease, and cancer [32]. Il‐8 is also consistently present in the SASP, which contributes to a state of chronic inflammation related to aging [31]. Although not statistically significant, a strong decrease in other inflammatory markers like IL‐1B, IL1Ra, and IL‐5 was also observed, demonstrating an overall improvement in the levels of systemic proinflammatory cytokines.

Compared to baseline, IL‐10, a known anti‐inflammatory cytokine, was also significantly reduced in the participants from the OS‐01 group. We believe that this change may be a reflection of the normalization of other inflammatory cytokines, balancing the amount of IL‐10. Regardless of the reduction observed in the levels of IL‐10, the values in the participants are in the normal range for healthy subjects [33]. IL‐10, despite its suggested beneficial effects in aging, has been found to limit protective responses against pathogens. It plays a detrimental role in chronic infections by impeding microbial clearance in both mice and humans [34]. Recent studies indicate that IL‐10 promotes the reactivation and replication of cytomegalovirus in latently infected mice [35]. Nevertheless, the links between inflammaging, IL‐10, and immune suppression remain largely unexplored [36]. Taken together, the overall reduction in the cytokine levels in the OS‐01 group suggests a potential correlation between improving the skin barrier function and protecting the body from aging‐induced increased systemic inflammation.

On the other hand, the control group presented an increase in the proinflammatory cytokines TNFα (*p* = 0.002) and IFN‐ɣ (*p* = 0.061). These two cytokines remained stable in the group treated with the OS‐01 formulation. This finding contrasts with a previous study using the control formula, which showed a significant reduction of IL‐1βB and IL‐6 [20]. Although the reason for the increase is unclear, it can be associated with the natural aging process and lifestyle differences that were not standardized in this study, like sleep, stress, diet, and exercise. Also, it is important to highlight differences in the study protocol when compared to the previous study [20]. The current study had a longer duration (12 weeks compared to 30 days in previous research). It was conducted in different countries (USA versus China), contributing to differences in genetic background among participants from each study and possible environmental influences. Moreover, while the control moisturizer improved the skin barrier function, it likely lacked the targeted anti‐senescence and anti‐inflammatory effects of the OS‐01 formulation.

The GlycanAge analysis provided additional insights into the potential influence of improved skin barrier function on systemic biological age and immune function by focusing on glycosylation patterns of IgG, a functional effector in immune regulation and chronic inflammation [37]. Different from epigenetic clocks, which measure DNA methylation changes primarily reflecting cellular aging mechanisms, GlycanAge directly captures changes in glycan structures on immunoglobulins that reflect systemic inflammation, a key driver of aging and age‐related diseases [38]. The observed stability in the glycan profile of OS‐01‐treated subjects, compared to a trend towards increased GlycanAge in the control group, corroborates what was observed in the instrumental analysis and systemic cytokine levels. These demonstrate that promoting a superior skin barrier provides a broad impact on aging processes beyond the skin. While skin health is sometimes viewed primarily as cosmetic, barrier health has a larger role in systemic health.

It is important to highlight that potential confounding factors such as lifestyle and diet can significantly influence inflammation and biological aging. Variables like physical activity, stress levels, sleep quality, and diet composition are known to impact both systemic inflammation and skin health [39]. While we conducted a lifestyle questionnaire with all participants and observed no changes in their lifestyle behaviors during the study (data not shown), it is important to note that behaviors were not standardized across participants. Future investigations should consider larger studies with a broader demographic, longer durations, and better control or stratification of lifestyle factors, which are necessary to generalize these findings and further investigate the mechanism by which skin treatment with OS‐01 topical formulation modulates systemic inflammation in humans.

In conclusion, our findings contribute to a growing body of evidence supporting the role of the OS‐01 peptide in addressing skin aging and promoting skin health. The observed improvements in skin health, which are reflected in reduced inflammation and systemic aging, demonstrate the importance of the skin in the intricate network of aging processes. Further research in this direction holds promise for advancing our understanding of skin‐centric interventions in the broader context of age management. It underscores the importance of adopting a holistic approach to pro‐longevity strategies.

## Author Contributions

Conception and design: A.Z., M.B., C.R.O., L.B.B., and J.L.C. Clinical trial execution: A.B.A., C.N.T. Data analysis and interpretation: L.B.B., A.Z., M.B., C.H., J.L.C. Drafting the article: J.L.C., A.Z., L.B.B., and N.H.O.H. Final revision: All authors. The authors read and approved the final manuscript.

## Ethics Statement

Before initiation, the study was approved by the Veritas Institutional Review Board (2022‐3038‐10 910‐5). The study was carried out in accordance with the principles set forth by the Declaration of Helsinki.

## Conflicts of Interest

Alessandra Zonari, Mariana Boroni, Carolina Reis de Oliveira, Lear Brace, and Juliana L. Carvalho are named as inventors of a patent directed at this invention, which is solely owned by OneSkin Inc. Alessandra Zonari, Mariana Boroni, Carolina Reis de Oliveira, and Juliana L. Carvalho are co‐founders of OneSkin Inc.

## Supporting information


**Table S1.** Inclusion and exclusion criteria.
**Table S2.** Ingredient list of experimental products.
**Table S3.** Mass spectrometry analysis of the human plasma from participants of the OS‐01 group (*n* = 27).

## Data Availability

The authors have nothing to report.
