# Supplementary material for: OS‐01 Peptide Topical Formulation Improves Skin Barrier Function and Reduces Systemic Inflammation Markers: A Pilot 12‐Week Clinical Trial
Source: J Cosmet Dermatol. 2025 Apr 7;24(4):e70169. doi: 10.1111/jocd.70169 (PMC11975214; doi:10.1111/jocd.70169)
Supplement: Supplementary file 1 — Table S1. Inclusion and exclusion criteria. Table S2. Ingredient list of experimental products. Table S3. Mass spectrometry analysis of the human plasma from participants of the OS‐01 group (n = 27). [file JOCD-24-e70169-s001.docx]

**Supplementary Table 1.** Inclusion and exclusion criteria for participants.

| **INCLUSION CRITERIA** | **1.** Females in good health.  **2.** Aged 60 years old or more, inclusive at the time of enrollment.  **3.** Able to read, understand and sign an informed consent form (includes HIPAA and State requirements).  **4.** Willing to be photographed and sign a photograph release form.  **5.** Willing and able to follow all study directions to perform study procedures as scheduled and to accept the restrictions of the study. |
| --- | --- |
| **EXCLUSION CRITERIA** | **1.** Participating in any other clinical studies  **2.** Subjects having an acute or chronic disease or medical condition, including dermatological problems, which could put her at risk in the opinion of the Principal Investigator or compromise study outcomes. Typical uncontrolled chronic or serious diseases and conditions which would prevent participation in any clinical trial are cancer, HIV/AIDS, diabetes, renal impairment, mental illness, drug/alcohol addiction  **3.** Subjects who are unlikely to be available for the duration of the study  **4.** History of allergic reactions, skin sensitization and/or known allergies to cosmetic ingredients, toiletries, sunscreens, etc  **5.** Immunocompromised subjects  **6.** Subjects who have initiated Hormone Replacement Therapy within the last three months preceding the screening visit  **7.** Women using oral contraception for less than three months before the Screening Visit or who have changed her contraceptive method within the three months before the Screening visit or planning to modify her contraception treatment within the duration of the study  **8.** Women known to be pregnant, lactating or planning to become pregnant within six months study start. Subjects who become pregnant during the study must inform the Principal Investigator immediately and will be excluded from the study  **9.** Individuals unable to communicate or cooperate with the Principal Investigator or staff. |

**Supplementary Table 2.** Ingredient list of experimental products.

| **Formulation** | **Ingredient list** |
| --- | --- |
| Control formulation: Atopalm® MLE Lotion | Water/Aqua, Glycerin, Propanediol, Myristoyl/palmitoyl Oxostearamide/arachamide MEA, Caprylic/capric Triglyceride, Cetearyl Alcohol, Glyceryl Stearate, Dimethicone, Carthamus Tinctorius (Safflower)Seed Oil, Portulaca Oleracea Extract, Polyglyceryl-10 Distearate, Sorbitan Stearate, Vitis Vinifera (grape) Seed Oil, Olea Europaea (olive) Fruit Oil, Hydrogenated Vegetable Oil, Tocopheryl Acetate, Phytosterols, Caprylyl Glycol, 1,2-Hexanediol, Allantoin, Arginine, Carbomer, Fragrance, Stearic Acid, Tropolone, Sodium Hyaluronate, Hydrolyzed Extensin |
| OS-01 formulation: OS-01 BODY | Water, Glycerin, Limnanthes Alba (Meadowfoam) Seed Oil, Sorbitan Olivate, Avena Sativa (Oat) Kernel Oil, Squalane, Prunus Domestica Seed Oil, Saccharide Isomerate, Cetearyl Olivate, d-Panthenol, Mangifera Indica (Mango) Seed Butter, Tremella Fuciformis (Mushroom) Extract, Tocopheryl acetate, Decapeptide-52*, Allantoin, Asiaticoside, Centella Asiatica Extract, Sodium Hyaluronate Crosspolymer, Ceramide NP, Anadenanthera Colubrina Bark Extract, Lepidium Sativum Sprout Extract, Genistein, Niacinamide, Glycerol, Bentonite, Cetyl Palmitate, Sorbitan Palmitate, Lecithin, Xanthan Gum, Polyglyceryl-10 Laurate, Polyglyceryl-6 Oleate, Sorbitan Oleate, Glyceryl Caprylate, Polysorbate 80, Alcohol, Caprylyl Glycol, Sorbic Acid, Citric Acid, Sodium Citrate, Pentylene Glycol, Potassium Sorbate, Ethylhexylglycerin, Caprylhydroxamic Acid, Tetrasodium Glutamate Diacetate, Phenoxyethanol. *OS-01 Peptide |

Supplementary Table 3 - Mass spectrometry analysis of the human plasma from participants of the OS-01 group (n=27).

| Time-point | Compound | Matrix | Measured Concentration  (ng/mL) |
| --- | --- | --- | --- |
| Baseline | OS-01 peptide | Human Plasma | BQL(< 1 ng/mL) |
| 12 weeks | OS-01 peptide | Human Plasma | BQL(< 1 ng/mL) |

BQL: below the quantification limit.
